# Four decades in the making: Collagen III and mechanisms of vascular Ehlers Danlos Syndrome

**DOI:** 10.1016/j.mbplus.2021.100090

**Published:** 2021-11-09

**Authors:** Ramla Omar, Fransiska Malfait, Tom Van Agtmael

**Affiliations:** a Institute of Cardiovascular and Medical Sciences, College of Medical, Veterinary & Life Sciences, University of Glasgow, G12 8QQ, UK; b Centre for Medical Genetics, Ghent University Hospital, Belgium; c Department of Biomolecular Medicine, Ghent University, Belgium

**Keywords:** Collagen, Ehlers Danlos Syndrome, Extracellular matrix, Endoplasmic reticulum stress, Disease mechanisms, Precision medicine

## Abstract

Vascular Ehlers Danlos (vEDS) syndrome is a severe multi-systemic connective tissue disorder characterized by risk of dissection and rupture of the arteries, gastro-intestinal tract and gravid uterus. vEDS is caused by mutations in *COL3A1*, that encodes the alpha 1 chain of type III collagen, which is a major extracellular matrix component of the vasculature and hollow organs. The first causal mutations were identified in the 1980s but progress in our understanding of the pathomolecular mechanisms has been limited. Recently, the application of more refined animal models combined with global omics approaches has yielded important new insights both in terms of disease mechanisms and potential for therapeutic intervention. However, it is also becoming apparent that vEDS is a complex disorder in terms of its molecular disease mechanisms with a poorly understood allelic and mechanistic heterogeneity. In this brief review we will focus our attention on the disease mechanisms of *COL3A1* mutations and vEDS, and recent progress in therapeutic approaches using animal models.

## Introduction

Mutations in collagen and collagen processing enzymes cause archetypical extracellular matrix (ECM) disorders such as osteogenesis imperfecta, Alport Syndrome and the Ehlers Danlos Syndromes [1], [2], [3]. These genetic multi-systemic diseases are rare, often debilitating and for many of them there are no treatments beyond management of symptoms. There is therefore an urgent need to unlock the molecular mechanisms of these extracellular matrix diseases from gene to patient level. Moreover, common non-coding and rare coding variants in collagen genes can be associated with and occur in sporadic forms of disease such as those coding for collagen IV in intracerebral haemorrhage [4], [5]. This is a powerful illustration that investigating rare genetic collagen disorders stands to inform on common disease forms. Moreover, as reduced collagen levels occur in many degenerative disorders [6], [7], and uncontrolled collagen deposition is a defining feature of fibrosis [8], increased understanding of collagen biology and genetic variants in collagen will have significant impact on addressing these major health problems. Recently technological advances with more refined animal models have provided windows of opportunity to address these major challenges. Here we will provide a brief review on vascular Ehlers Danlos Syndrome (vEDS) and the mechanisms of *COL3A1* mutations that have uncovered mechanistic heterogeneity and potential mechanism-based treatments.

## Ehlers-Danlos syndromes

The Ehlers-Danlos syndromes comprise a genetically heterogeneous group of heritable connective tissue disorders that share several characteristics such as soft and hyperextensible skin, abnormal wound healing, easy bruising and joint hypermobility [2]. Additional clinical features that differ among the EDS subtypes include fragility of soft tissues, blood vessels and hollow organs, and involvement of the musculoskeletal system. Mutations in genes coding for fibrillar collagens (type III and V and to a lesser extent type I) are found in the more prevalent forms of EDS, such as vascular and classical EDS, but additional rare EDS subtypes are caused by mutations in genes coding for a series of matrix-related molecules. Currently, 13 distinct EDS subtypes are recognized, with mutations found in 20 different genes [9].

With an estimated prevalence of 1:20,0000–50,000, the autosomal dominant vEDS (OMIM # 130050) is considered one of the most severe EDS subtypes as affected individuals are at risk for life-threatening ruptures of medium- and large-sized arteries, the gastro-intestinal tract, the gravid uterus and other internal organs such as the liver or the spleen. Consequently, life expectancy of vEDS patients is reduced by multiple decades to ∼50 years [10]. Other features include easy bruising, thin and translucent skin with increased venous visibility, acrogeria, characteristic facial features (large eyes, periorbital pigmentation, small chin, sunken cheeks, thin nose and lips and lobeless ears), spontaneous pneumothorax, talipes equinovarus, congenital hip dislocation, small joint hypermobility, tendon and muscle rupture, gingival recession and gingival fragility and early-onset varicose veins.

## Collagen III, processing and fibril formation

Type III collagen is the second most abundant fibrillar collagen and is associated with collagen I in all soft tissues, in particular those with elastic properties including dermis, blood vessels, and gastro-intestinal tract where it can make up 10–30% of collagen content [11]. Collagen III provides structural support to tissues, influences cell behaviour and function through binding cell surface receptors such as integrin (e.g. α_1_β_1_, α_2_β_1_), and plays important roles in wound healing, angiogenesis, development and cell differentiation [12], [13]. Other binding partners include other ECM molecules such as proteoglycans (e.g. Serpin F1, decorin), collagens (e.g collagen I, V) and fibronectin [12]. Like all mammalian collagens, it is a large protein of >300 nm in length with a molecular weight >300 kDa, which has severely hindered detailed structural analysis. While diffraction-based approaches and those using short peptides provided critical fundamental insight into the basis of D-banding and triple helix structure and stability [14], [15], the impact of sequence variation in a full length-molecule has only very recently been investigated using atomic force microscopy [16]. This revealed, surprisingly and in contrast to data from short peptides, that proline content did not correlate with local flexibility of the protein. It also confirmed that collagen III has varying flexibility determined by sequence variation, and that the N-terminal region has the highest structural flexibility, in particular the matrix metalloprotease (MMP) binding site, which plays an important role in ECM remodelling, causing the high bending flexibility of this region of collagen III [16]. Collagen III adopts a flexi-rod structure in which in silico domain mapping suggested that the rigid domains align with functional domains such as the two hemostasis domains [12]. One of these domains contains a single binding motif for von Willebrand Factor [17] that mediates platelet adhesion, with bruising being a very well characterised feature of vEDS [12]. The second hemostasis domain binds the platelet receptor glycoprotein VI [12], [18] and integrin α_2_β_1_ that are involved in platelet binding and hemostasis [19]. The biding sites for integrin α_2_β_1_, heparin, vWF, decorin and fibronectin are also involved in angiogenesis [12].

**Fig. 1:**
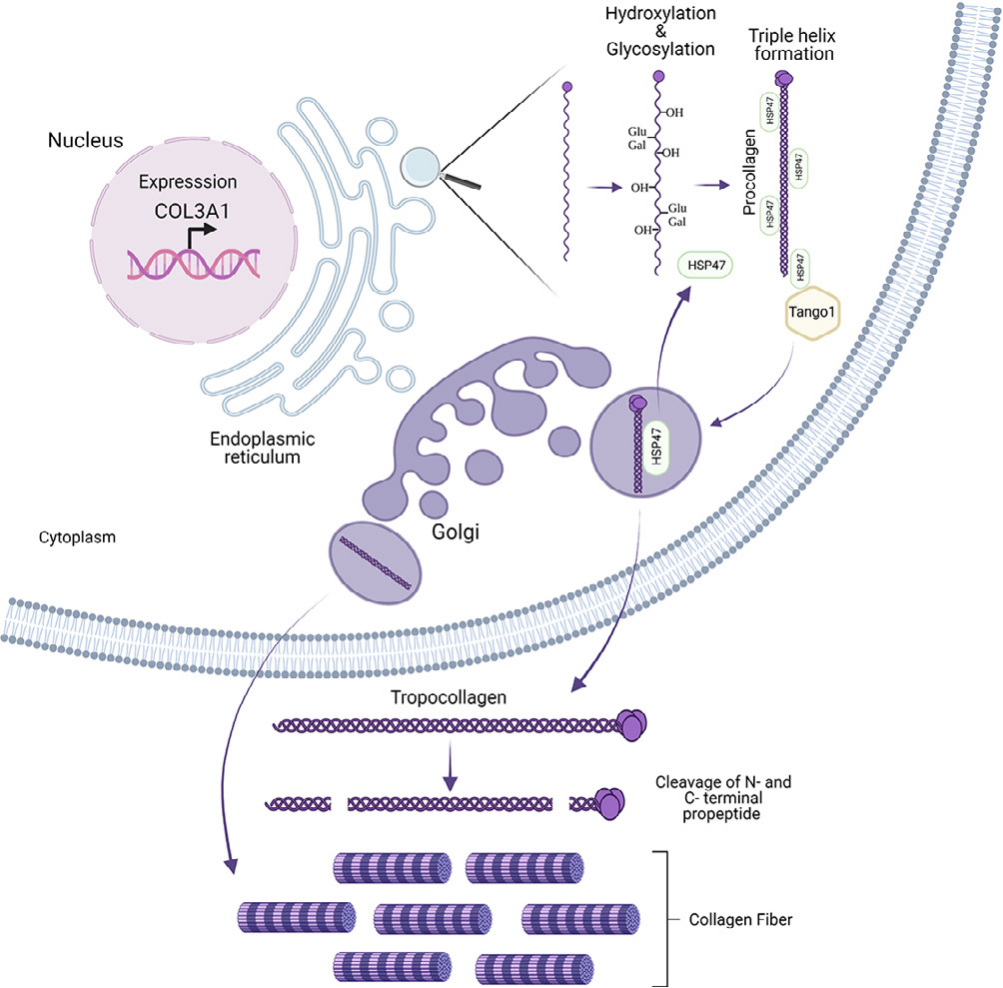
Overview of collagen III protein domain structure and processing by N- and C-proteinases to generate triple helical collagen III that forms heterotypic fibrils with collagen I. Collagen secretion contains several key stages including expression and folding in the ER where the nascent α chains are post-translationally modified by hydroxylation of proline and lysine residues, as well as of glycosylation and galactosylation of hydroxylysines. In the ER the collagen specific chaperone HSP47 binds to collagen III and plays key roles in collagen folding and transport to Golgi by binding TANGO1 and collagen. HSP47 is recycled from the Golgi back to the ER. In the Golgi and ECM, collagen III undergoes cleavage of the N- and C-propeptide, and the triple helical collagen III then forms heterotypic fibrils with collagen I.

Collagen III forms a homotrimer, containing three α1(III) α-chains encoded by the *COL3A1* gene on chromosome 12. The α-chains comprise of three structural domains with N-terminal and C-terminal propeptides that flank a triple helical collagenous domain (Fig. 1). This collagenous domain is characterized by the collagen Gly-Xaa-Yaa repeat in which every third residue is a glycine, and Xaa and Yaa can be any amino acid but are often proline and hydroxy-proline. These glycines are critical for triple helix formation as only the smallest amino acid can fit in the internal space of the triple helix, and are most frequently affected in collagen disorders due to missense mutations [1], [20]. In a very elegant and landmark paper, David Hulmes and colleagues revealed that the C-propeptide has the shape of a flower with a stalk, base and three petals [21]. The petals are critical for α-chain recognition to commence trimerization, whereas the base then stabilizes the trimer [21]. Remarkably despite identical amino acid sequences, one α-chain adopts a different conformation to enable efficient packing of the three α-chains, also providing insight into the basis of α-chain composition for respective collagen homo- and heterotrimers [21].

Like all secreted and transmembrane proteins, collagen III is folded in the endoplasmic reticulum (ER) (Fig. 1). In the ER collagen α-chains undergo extensive post-translational modification that are required for its folding and solubility. For triple helix formation to occur peptidyl prolyl cis–trans isomerases such as cyclophilin B and FK506 binding protein 22 (FKBP22), and FK506 binding protein 65 (FKBP65) convert all proline residues to the trans form [22]. FKBP22 shows substrate specificity as it binds collagen III (and VI and X) but not collagen I, II or V [23], but appears to act post prolyl hydroxylation (see below) [24], and mutations in or absence of FKBP22 cause Kyphoscoliotic EDS [2]. These prolines can then be hydroxylated by prolyl 4-hydroxylase (P4H) and prolyl 3-hydroxylase (P3H) that provide thermal stability to the triple helix [25]. P4Hs hydroxylate prolines in the Yaa position of the Gly-Xaa-Yaa repeat and are tetramers consisting of 2 α and 2 β units in which the enzyme PDI (protein disulphide isomerase) is the β sub-unit. Mammals express three P4H isoforms, P4Ha1-3, and they hydroxylate almost all prolines in the Yaa position [26]. The clinical and functional importance of prolyl 3-hydroxylation became apparent from mutations in the *CRTAP* gene (cartilage-associated protein) that cause recessive forms of osteogenesis imperfecta [1]. CRTAP forms a complex with cyclophilin B and P3H1 to hydrodroxylate prolines, and mammals express three P3H isoforms (P3H1-3). Interestingly while in the network forming collagen IV, 10% of the total hydroxyl-proline can be 3-hydroxylated, in fibrillar collagen only a few 3-hydroxylated prolines occur (e.g. 1–2 residues for collagen I, 3–6 in collagen V and XI) [26], [27]. Remarkably, and indicating collagen specificity, in mammals collagen III does not undergo 3-proline hydroxylation although in chicken it does, indicating this modification has been lost in evolution [27]. Hydroxylation of lysine residues at the Yaa position of the Gly-Xaa-Yaa repeat is mediated by lysyl hydroxylases (LH1-LH3), important for formation of intermolecular collagen crosslinks, and these lysines can then be glycosylated and galactosylated by galactosylhydroxylysyl-glucosyl transferase and galactosyl transferase, respectively [28]. The importance of lysyl hydroxylation for EDS is clearly illustrated by the fact that mutation in the *PLOD1* gene, encoding LH1, cause kyphoscoliotic type 1 Ehlers-Danlos syndrome [2]. Similarly to 3-prolyl hydroxylation, the levels of hydroxylated lysine is much higher in collagen IV as compared to collagen I and III, and LH3 does not hydroxylate lysine in collagen I and III [24]. In addition, LH1 and LH2 also have substrate specificity with LH1 hydroxylating collagens I and III, but not collagen II, IV and V. For a more in-depth review on post-translational modification, folding of collagen and its quality control please see these excellent reviews [1], [3], [24], [26], [28], [29].

Concomitantly with these modifications, three Proα1(III)-chains associate in the ER via their C-propeptides which then initiates triple helix formation in a zipper-like fashion in a C- to N-terminal direction. Collagen folding requires chaperones including the collagen specific chaperone heat shock protein 47 (HSP47) that binds and stabilizes collagen in the ER and during transit to the *cis*-Golgi, where HSP47 dissociates and is recycled back to the ER [30] (Fig. 1). Recent data uncovered that HSP47 has multiple roles in collagen processing, some of which can be collagen type specific, including in collagen secretion, lateral assembly, and triple helix formation [31]. HSP47 aids the translocation of collagen from the rough ER via the ERGIC (ER Golgi Intermediate Compartment) to the Golgi for secretion to the ECM, by acting as an anchor between the TANGO1 protein and collagen to allow packaging of collagen in secretory vesicles (Fig. 1) [32]. For an in depth review on the secretion of large cargo proteins we refer the reader to [33].

Folded collagen in the Golgi then undergoes cleavage with cleavage of the C-propeptide by procollagen C-proteinases (which are identical to the BMP-1/tolloid proteinase) that is required for fibrillogenesis (Fig. 1) [22]. The N-propeptide is cleaved by procollagen N-proteinases, that are identical to the A Disintegrin And Metalloproteinase with Thrombospondin motifs (ADAMTS) proteinases ADAMTS 2, ADAMTS 3 and ADAMTS 14 proteinases (Fig. 1) [22]. For collagen III this cleavage is not always complete [34]. This ultimately results in the formation of proteins that have uninterrupted triple helices flanked by telopeptides that self-assemble into fibrils (Fig. 1). These fibrils are composed of more than one collagen type, which is referred to as heterotypic fibrils; for example collagen III associates with type I collagen forming heterotypic type I:III collagen fibrils.

## Collagen III mutations in vEDS

vEDS is caused by heterozygous mutations in the *COL3A1* gene and to date >500 *COL3A1* mutations have been reported (https://www.le.ac.uk/genetics/collagen/). About 65% of mutations substitute glycine residues in the canonical triplet repeats of the proα1(III) triple helix domain, and about 25% are splice site variants that result in in-frame exon skipping. A small proportion results in short in-frame deletions or insertions (Table 1). Because procollagen III is a homotrimer, the synthesis of an equal amount of normal and mutant α1(III) chains results in seven-eighths of the homotrimers being abnormal (containing either one, two or three mutant α-chains). In 2001, Schwarze and coworkers reported the first *COL3A1* haploinsufficiency mutations [35], and since then ∼5% *COL3A1* mutations have been identified that lead to introduction of a premature termination codon and mRNA instability [10].

**Table 1:** *COL3A1* mutations in vEDS. The data presented for the different types of mutations is based largely on [10], [37].

Mutation type	Prevalence	Effect on protein	Age at diagnosis	Genotype-phenotype
Glycine mutations	65%	Collagen III structural abnormality	∼34 years	Severity is increased with substitution of glycine with larger charged amino acids. Most severe phenotype after splice site mutation
In frame splice site mutations	25%	Collagen III structural abnormality	∼25 years	Most severe phenotype and lowest median age of survival
Null mutations	5%	∼50% Reduction of collagen III	∼46 years	Mild phenotype and high median age of survival
Other missense mutations (insertions, deletions, C- and N-terminal mutations etc.)	5%	Collagen III structural abnormality	∼45 years	Mild phenotype and high median age of survival

In terms of genotype-phenotype correlations, null-mutations are associated with a delayed onset of complications by two decades and a reduced penetrance, and complications seem to be limited to vascular events [10], [36]. Other genotype-phenotype correlations recently emerged from studies in larger cohorts of vEDS patients [10]. Individuals with in-frame exon-skipping splice site variants tend to have the lowest median survival, followed by glycine substitutions within the α1(III)-triple helical domain by a bulky residue (arginine, aspartic acid, glutamic acid, valine), while those with small residue substitutions for glycine (alanine, serine, cysteine) present milder phenotypes. Missense variants in the C-propeptide of the proα1(III) chain, and substitutions in the Xaa and Yaa-positions in the triple helical domain can be associated with mild signs of vEDS and arterial fragility [37]. Among the latter group, substitutions of glutamic acid by lysine were recently shown to be associated with a skin phenotype that is more similar to that seen in classical EDS (skin hyperextensibility, delayed wound healing, joint hypermobility), combined with gastro-intestinal and vascular fragility [38]. The crystal structure of the C-propeptide also provided insight that most severe mutations in this domain are located at the petal-base region interface, the petal tips and the base region, and affect intrachain disulfide bonds, interchain interactions or stability of the hydrophobic core [21]. Mutation at the surface not involved in these interactions and thus folding or trimerization tended to be milder mutations [21]. A few individuals have been identified with bi-allelic *COL3A1* variants; they had a severe vEDS phenotype, associated with neuronal post-migrational disorder (polymicrogyria) [39], [40], [41], [42]. It should however be noted that the molecular basis for these genotype-phenotype correlations remains poorly defined.

## Collagen III variants in the general population and other diseases.

To date (September 30, 2021) 1904 variants in *COL3A1* have been submitted to the Clinvar database (https://www.ncbi.nlm.nih.gov/clinvar), of which 489 have been classed as pathogenic and 22 as likely pathogenic. For those for which a diagnosis was provided this included vEDS, polymicrogyria with VEDS, or familial thoracic aortic aneurysm and aortic dissection. Other variants, including those affecting glycines and a frameshift mutation, have also been reported in sporadic thoracic aortic aneurysm [43], illustrating the importance of collagen III in sporadic disease and that investigating rare genetic forms may inform on and apply to mechanisms of at least some cases of sporadic disease.

The intolerance to mutations in *COL3A1* is also apparent from data in the gnomAD database (https://gnomad.broadinstitute.org/) containing exome sequence information of 125,000 individuals (individuals with severe paediatric disease and their first degree relatives are excluded) [44], which shows that to date 516 missense variant have been observed, compared with an expected 851.7 variants based on sequence length (Z = 4.09, observed/expected ratio = 0.61 (90% confidence interval: 0.56–0.65). Strikingly, compared to the expected ∼94 loss of function variants, only 4 are reported. These data clearly illustrate the pathogenic impact of variants in *COL3A1* and intolerance to loss of function mutations. Examination of the data does reveal additional potential loss of function alleles are present with 3 variants predicted to generate a stop codon, 4 frameshift variants, 3 spice acceptor variants and 2 splice donor variants. Furthermore 7 missense variants have been classed as pathogenic/likely pathogenic, of which 6 affect glycine residues distributed across the triple helix, and one Proline variant in the N-propeptide. Other missense variants that remain unclassified or have conflicting classification are distributed across the protein. Overall, the potentially pathogenic variants in gnomAD illustrate the variable clinical severity of the disease, while those many unclassified variants illustrate our limited knowledge regarding the effect of variants on collagen biology. This directly impacts on patient management and illustrates the need for molecular analysis of variants to aid their stratification and increase much needed understanding of the genotype-phenotype relation.

Other diseases. Bi-allelic variants in *COL3A1* have been identified in patients with brain defects with and without vEDS [13], [40], [42]. The patients can develop frontoparietal polymicrogyria, with migration defects, and aneurysmal brain haemorrhage, that were similar to those observed in patients with mutations in G protein-coupled receptor 56 (GRP56) that binds collagen III. To date the mechanism(s) remain unclear but these data do support a role of collagen III in neuronal migration.

Given the vascular defects *COL3A1* mutations cause in vEDS, it is no surprise that over the last 30 years multiple sequencing and genetic association studies have been undertaken to interrogate if mutations occur in *COL3A1* or if common variants are a risk factor for vascular diseases, respectively. This revealed *COL3A1* mutations have been detected in families with abdominal and thoracic aorta aneurysm [45], [46], [47], but also in apparent sporadic abdominal aortic aneurysm in which an altered collagen I/III ratio was also observed [46], and thoracic aorta aneurysm [43]. However, no association was found for common variants in genome wide association studies for aneurysm formation [48]. This supports a role for rare coding-variants with a large effect in a small percentage of cases but not common (non-coding) risk variants with a small effect size.

Genetic analysis of three common variants in *COL3A1* in a cohort of Chinese stroke patients suggested an association with stroke recurrence and prognosis [49]. However, no association with stroke risk was detected in a large genome wide meta-analysis covering ∼500,000 patients [50]. While this may reflect difference in ethnicity of study population, and different clinical trait (e.g. stroke prognosis versus occurrence of stroke), given the limited analysis it is important to independently replicate these findings. Other diseases including fibrotic disorders, cardiomyopathy, diabetic nephropathy, cancer (e.g. glioma, breast cancer) have been associated with collagen III as they are characterised by altered collagen I and III levels (For a more in depth overview see [13]).

Finally, analysis of the Tsk2/+ mouse model (Table 2) also suggests a role in systemic sclerosis. In this model, a missense mutation in the N-propeptide of α1(III) results in a phenotype similar to systemic sclerosis [51]. While genome wide association studies have not identified genetic association with *COL3A1* [52], this does not rule out that some rare coding variants in the N-propeptide may contribute to some cases of systemic sclerosis. In depth sequencing analysis of a large patient cohort would address this mutation and may further establish the clinical importance of N-propeptide variants and collagen III.

**Table 2:** *Col3a1* mouse models. Only original references are provided except in case whereby there is some debate regarding the nature of the mutation.

Model	Genotype-Method	Phenotype	Reference
*Col3a1*^−/−^	Targeted deletion *Col3a1* promoter and exon 1	5% survival after birth, major skin lesions, vascular rupture, abnormal fibril organization.	[53]
*Col3a1*^+/−^		Mild phenotype, normal life span, aortic lesions, reduced collagen III, elevated MMP9 levels.	[55]
*Col3a1*^+/−^ (*Col3a1^m1Lsmi/+^)*	Targeted deletion (*Col3a1* promoter and exon 1–39)- in frame deletion exon 33–39 upon subsequent analysis.	Mild phenotype, aortic dissection, reduced collagen III. 30% lethality at 3 month	[56]
*Col3a1*^Tg-G182S/+^	Overexpression of *Col3a1* transgene harbouring glycine substitution (Gly182Ser)	Thin and fragile skin, open wounds, vascular fragility, reduced collagen III, abnormal fibril organization	[57], [58]
*Col3a1*^G209S/+^	CRISPR glycine substitution	Vascular phenotype, sudden death due to aortic rupture, abnormal fibril organization, median survival 400 days	[51]
*Col3a1*^G938D/+^	CRISPR glycine substitution	Vascular phenotype, sudden death due to aortic rupture, abnormal fibril organization, median survival 45 days.	[51]
*Tsk2/+ (Col3a1 C33S/+)*	ENU mutagenesis, missense mutation in N-terminal propeptide	Tight skin (increased ECM deposition), thick collagen fibrils, model of systemic sclerosis	[60]

## Animal models

Investigating animal models has been and will be critical to disentangle the disease mechanisms of vEDS and developing more refined animal models has underpinned recent mechanistic insights. The first generated *Col3a1^−/−^* mouse model had skin lesions with open wounds, and analyses of the heart and aorta showed a significant reduction or absence of collagen fibrils in the media of the aorta [53] (Table 2). The wall of arteries consists of an endothelium, that forms the tunica intima and is surrounded by layers of vascular smooth muscle cells (vSMC) that form the tunica media. The media is surrounded by the adventitia which contains several cell types including fibroblasts and is rich in ECM. In between the vSMC layers are elastic lamella and collagen fibrils, that are secreted by the vSMC [54] into the ECM and provide elasticity and tensile strength, respectively. Interestingly, in the adventitia of mutant mice, the collagen fibrils in the adventitia had larger diameters [53]. As the majority of the adventitial collagens is collagen I, this provided *in vivo* evidence that collagen III regulates collagen I:III fibril formation. Surprisingly based on the severity of vEDS, *Col3a1^+/−^* mice have a normal life span and showed no overt phenotypes [53], [55]. However, reduced collagen III content in aorta and bowel and reduced wall strength was detected, and aortic lesions progressed with age, indicating biomechanical differences [55]. These data were confirmed in a second mouse model which was generated by a spontaneous mutation [56]. While the exact nature of the mutations remains debated with reports of both a null allele and in-frame deletion leading to a truncated transcript [56], [57], heterozygous mutant mice present with aortic dissection, and increased lethality at 4 months of age and therefore recapitulate vEDS phenotypes (Table 2). It should be noted that these mice do not develop skin, gastrointestinal defects nor is cardiac function altered [56].

In contrast to these mice with deletions, which represent the minority of mutations identified, recently, three mouse models have been reported that express *Col3a1* glycine mutations. *Col3a1^Tg-G182S^* mice transgenically express a *Col3a1* Gly182Ser mutation and develop fragile thin skin with severe open wounds, and fragility of the aorta, although no spontaneous arterial or intestinal rupture was observed [58]. These mice therefore phenocopy some vEDS features, although it should be noted that they express collagen III at supra-physiological levels. Most recently CRISPR was used to generate *Col3a1^G209S/+^* and *Col3a1^G938D/+^* mice that exhibited vascular phenotypes found in vEDS patients including sudden fatal aortic ruptures, and the more C-terminal mutation had a more severe phenotype with lower survival rate (Table 2) [51]. These data support the human genetic data of more severe phenotypes being associated with C-terminal triple helix mutations.

Finally, the Tsk2/+ (tight skin 2) model was identified from a ENU mutagenesis screen in the 1980s and has been characterised for its skin phenotype that resembles systemic sclerosis with tight skin, increased collagen I deposition and fibrosis, and thicker collagen fibrils [51]. As observed in patients, the mice also develop auto-antibodies [59], and these phenotypes are due to a missense mutation in the N-terminal pro-peptide of collagen III (*Cys33S*er) [51]. In contrast to vEDS the skin of this mouse model was thickened, and the mutations lead to increased collagen III levels [51], which could explain the very different clinical phenotype. To date, no analysis of vascular defects has been reported. The mechanism of this mutation is intriguing as transgenically increased wild type collagen III levels in another mouse model does not cause overt defects [58]. Does it have additional effect beyond increased collagen levels? This nicely illustrates the lack of understanding of how missense mutations in collagen III act, and the need for more detailed analysis to unpick this complexity.

## Disease mechanisms of vEDS

### Extracellular and intracellular defects

While the association between collagen III and vEDS has been known for more than four decades [61], [62], the molecular and cellular mechanisms by which *COL3A1* mutations cause vEDS remain poorly understood. A key approach to help bridge this gap has been the biochemical and cellular analysis of dermal fibroblast cell cultures from vEDS patients [63]. They greatly express collagen III, are physiologically relevant given the skin defect in patients, and may shed some light on the vascular defects as fibroblasts are important in the adventitia of blood vessels. Thus, while dermal fibroblasts may not recapitulate vSMC defects, they may be informative for adventitial fibroblasts.

**Fig. 2:**
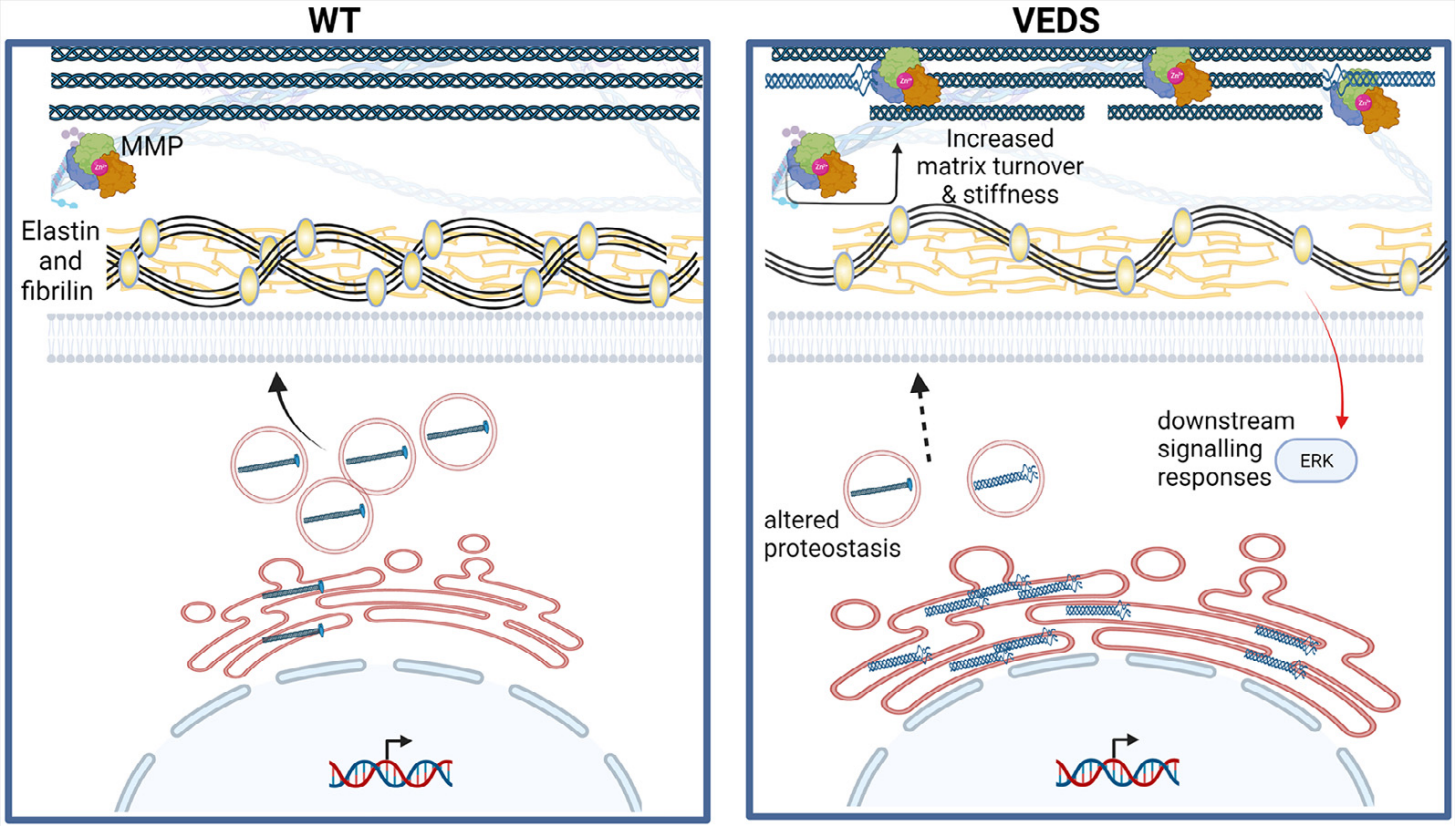
Potential disease mechanisms. Compared to wild type (WT), nonsense *COL3A1* mutations lead to reduced secretion of wild type collagen III and extracellular levels of wild type collagen III causing fibrillar defects (interrupted fibrils). Missense mutations lead to expression of mutant collagen (distorted helix) that can be secreted and/or lead to intracellular accumulation of collagen in the ER, that can result in altered proteostasis indicated by ER enlargement. Therefore, both altered proteostasis and reduced secretion may occur simultaneously. The impact of *COL3A1* mutations ion the ECM include reduced collagen III levels, and presence of mutant collagen III that may lead to disruption of the collagen network. This can be coupled with matrix turnover and degradation mediated, at least in part, by higher MMP levels. Furthermore, elastin defects occur with reduced levels of fibrillin 2. These ECM defects have been proposed to cause via an as yet unknown mechanism activation of ERK signalling. While ER enlargement is regularly observed, the nature of the altered proteostasis remains poorly defined. The multisystemic nature of vEDS also leaves the door open for cell and mutation specific effects, which represent an important gap in our knowledge.

Initial investigations by Pope and colleagues uncovered significantly reduced collagen III secretion in dermal fibroblasts from patients [61] and since then lower extracellular collagen III levels are accepted as a defining feature of vEDS, also observed in tissues such as aorta and skin of mouse models harbouring missense mutations [51], [57]. The reduced extracellular levels can reflect reduced collagen III expression as seen with non-sense mutations (Fig. 2) but *COL3A1* glycine mutations can also cause a reduction in other collagens, including collagen I [58]. Mechanistically, it is possible that the reduced bioavailability of collagen III can delay fibrillogenesis and/or leads to degradation of collagen I, but may also reflect reduced secretion of collagen I [64], although this needs to be further determined. Given the direction of the triple helix formation one can predict a more severe effect of C-terminal mutations. This is supported by an almost complete failure of fibroblasts harbouring variants (glycine mutations and exon-skipping variants) at the C-terminal end of the triple helix to secrete type III collagen (Fig. 2). The non-secreted type III procollagen can be sequestered in the rER (which can appear dilated) where it is overmodified and very slowly degraded [63], see also below. In contrast, there is less clear evidence of intracellular retention for N-terminal mutations, suggestive of rapid intracellular degradation [65]. As mentioned above, for C-propeptide mutations milder mutations were not predicted to interfere with triple helix folding or trimer formation, in contrast to severe mutations (the latter located at the petal-base region interface, the petal tips and the base region) [21].

Electron microscopy analysis on skin samples of patients revealed altered collagen fibril diameter [66], [67], which was also observed in mice deficient for *Col3a1* and those that transgenically express a *Col3a1* glycine mutation with variable fibril diameters and a preponderance for thicker fibrils [53], [58]. In patient skin biopsies more C-terminal triple helix mutations have been associated with smaller collagen fibril diameters (65–80 nm versus 93 ± 7.5 in wild type) [68], in contrast to a more variable fibril diameter (85–120 nm) for N-terminal triple helix mutations [67]. This is supported by the observation that collagen fibrils with a higher collagen III:I ratio tend to be thinner, and that fibrils with more collagen I have a larger diameter [69], [70], indicating incorporation of collagen III limits fibril diameter. Interestingly, the association of altered collagen I:III ratio, reduced collagen III or increased collagen I levels with sporadic aortic aneurysm formation [71] and the recent important identification of *COL3A1* mutations in patients with sporadic thoracic dissection [43], underscores the clinical relevance of collagen III in both genetic and sporadic vascular disease including aortic aneurysms. It also supports that increased mechanistic insight into vEDS can represent a gateway to understanding the molecular basis of some cases of sporadic aneurysm formation.

Extracellular matrix stability and function are controlled by the activities of the ECM proteases such as matrix metalloproteinases (MMPs). Interesting in this regard is the increased expression of MMP9 in the aorta of *Col3a1^+/−^* mice [72], [73]. In addition, enhanced sensitivity of secreted collagen III to proteases leading to reduced levels has also been put forward as a mechanism [74]. MMP levels have been associated with many ECM disorders and increased MMP activity could therefore lead to further damage to an already weakened ECM caused by secretion of mutant protein and/or reduced extracellular collagen III (Fig. 2). However, the involvement and relative contribution of these processes to the disease mechanisms, the cell types and tissues in which they occur, and the extent to which they apply to all mutations remain unclear.

These matrix defects can be accompanied by dilation of the ER in the skin of patients [63], [66], [67], as well as in the vasculature of mice with a glycine mutation [51] suggesting collagen III intracellular accumulation. However, this has not been consistently observed as transgenic mouse models did not show any signs of ER stress in dermal fibroblasts [58]. Accumulation of mis- or unfolded protein can lead to ER stress (for a detailed review on ER stress, we refer the reader to these excellent reviews [75], [76]) a response that aims to restore ER homeostasis by upregulating the expression of ER-resident chaperones to increase protein folding capacity and protein degradation pathways, while reducing global protein synthesis. However, chronic activation of the Unfolded Protein Response (UPR) can activate apoptotic pathways and has been associated with numerous disorders [76], [77]. Importantly, elegant analysis has revealed that for metaphyseal chondrodysplasia type Schmid (OMIM # 156500), caused by mutations in *COL10A1* coding for collagen X, the ER stress and not the matrix defects are the proximal molecular mechanism [78]. Moreover, ER stress has also been associated with other collagen and ECM disorders such as osteogenesis imperfecta due to mutations in collagen I and *COL4A1/2* related disorders [3], [76]. The evidence for intracellular effects and UPR activation caused by intracellular retention is less clear for vEDS, which may reflect allelic heterogeneity, potentially coupled with cell type dependent mechanisms. vEDS analysis of dermal fibroblasts has indicated some signs of ER stress, including expression of C/EBP homologous protein (CHOP; a marker of chronic UPR) and activation of apoptosis [79], [80], although a systematic in-depth analysis of the UPR was not reported. Elegant work by Bächinger and colleagues also showed that in a bacterial expression system, glycine *COL3A1* mutations caused a delay in protein folding [81]. Recent RNA Seq analysis on fibroblasts indicated perturbation in genes that help maintain ER homeostasis, including downregulation of those involved in proteasomal degradation, formation of disulphide bonds during protein folding, and heat shock proteins that act as chaperones. However, it should be noted that activation of the classical UPR was not reported [82], [83] (Fig. 2). Overall, this describes in dermal fibroblasts an environment whereby *COL3A1* mutations can affect ER homeostasis via accumulation of collagen III, with dysregulated proteasomal degradation and a multi-faceted cellular response to these mutations.

The absence of a classical UPR was also observed in dermal fibroblasts of transgenic *Col3a1^Tg^*^-G182S/+^ mice with no increased dilation of the ER or UPR activation [58]. This could theoretically reflect an allelic effect as ER dilatation has been related to mutation severity with more C-terminal mutations having a larger ER and reduced collagen III secretion [67]. In addition, increased expression of wild type *Col3a1* was used as control [58] which could have increased chaperone levels by elevating protein folding demand as daily flux of collagen expression and secretion are matched with that of ER chaperones [84]. In the mouse models with a glycine mutation in the endogenous *Col3a1* genes ER enlargement was observed by electron microscopy in the adventitial fibroblasts but no ER stress or UPR activation was detected via RNA Seq analysis of aorta [51]. It remains unclear if there were any signs of altered proteostasis in the absence of a classical UPR/ER stress, as observed in patient fibroblasts [82], [83]. Furthermore, the bulk RNAseq on entire tissue such as aorta may miss cell-specific mechanisms in adventitial fibroblasts, which showed signs of ER dilatation (a marker of ER stress), compared to vascular smooth muscle cells.

Taken together this supports the existence of mechanistic heterogeneity within vEDS due to the allelic nature of the mutation with potential cell and tissue specific responses to the mutations, which we also observed in *Col4a1* mutant mice [85]. While the matrix defects due to reduced levels of collagen III and/or the presence of mutant collagen III are well-established, the impact and presence of protein misfolding is much less clear. These mutually non-exclusive mechanisms may both contribute to the disease but currently it remains unclear if the ER stress is cause or effect and, even if present, contributes to the pathogenesis. A systematic detailed molecular analysis of an allelic series of mutations across different disease-relevant cell types is needed and would fill this important knowledge gap.

### Downstream mechanisms

While reduced collagen III levels in the ECM, the secretion of mutant collagen III and ER stress represent upstream molecular mechanisms, their downstream effects and how they cause vEDS remain poorly defined. The mutations cause vascular fragility associated with increased circumferential wall stress but unchanged arterial stiffness, measured as pulse wave velocity, and elastic properties of the vascular wall (Young’s modulus) [86]. Morphologically, the mutations lead to reduced intima-media thickness [51], [86] but do not affect internal diameter [86]. While mouse models support a contribution of reduced collagen levels [53], given the ECM composition of the vascular wall whereby collagen accounts for ∼13–18% [87], other factors likely play a key role in this vascular dysmorphology. The nature of these remains unclear but alterations in cell behaviour and signalling (see also below) are likely candidates. Whether these are in response to the reduced levels of collagen III, secreted mutant collagen III and/or proteostasis disruption is an important knowledge-gap. The biomechanical impact of the *COL3A1* mutations on tissue fragility is not limited to the vasculature. Patients have thin, fragile skin, which splits easily and heals slowly with formation of thin scars, which is also present in the mouse models that develop skin fragility and open wounds [53], [58] However, this phenotype remains under-investigated in the different animal models and represents an important gap in our understanding that needs to be bridged.

To better understand the biological processes by which these mutations cause disease, Chiarelli and colleagues performed transcriptomics analysis of a small allelic series of dermal fibroblasts with *COL3A1* mutations (two glycine and one in frame splicing mutation) [82]. This revealed the cell cycle was the most dysregulated pathway with downregulation of genes (e.g. cyclin dependent kinases) involved in cell division, DNA replication, telomere organization etc. [82]. This impact on the cell cycle may be downstream of altered DNA damage responses that interestingly cross-talk with ER stress and proteostasis [88] and/or ECM defects as integrin mediated cell signalling influences cell proliferation [89]. The *COL3A1* mutations also affected the mRNA levels of several major ECM components that provide ECM structural integrity. This includes reduced levels of FBN2 (fibrillin 2) that plays a key role in providing elasticity along with elastic fibers in many tissues, in particular blood vessels (Fig. 2), and modulates TGFβ signalling, which is well known to be involved in vascular connective tissue disorders such as Marfan Syndrome, by binding LTBP [90], [91], [92]. Interestingly increased serum levels of TGFβ have been observed in vEDS, but no evidence of increased TGFβ mediated signalling was detected in fibroblasts of patients [93]. Together with the disorganization of elastin (ELN) and elastin microfibril interface-located proteins (EMILINs) that provide elasticity in blood vessels and skin tissue [94], many of these affected mRNAs could contribute to the vascular fragility in vEDS (Fig. 2).

**Fig. 3:**
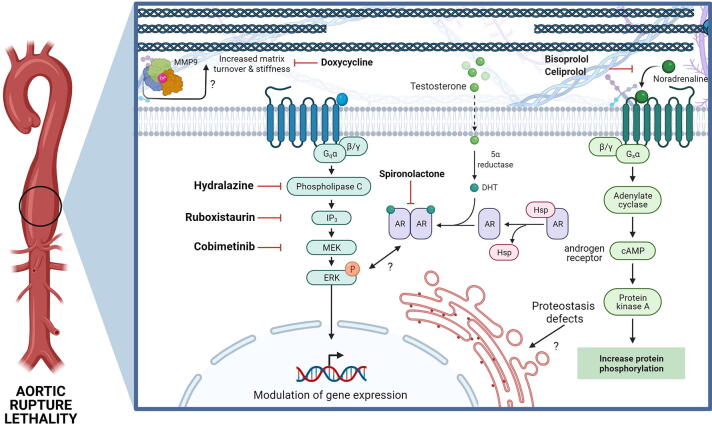
Therapeutic approaches for VEDS have focused on addressing the aortic rupture. In particular β targeting adrenergic signalling via celiprolol, which has been used in clinical trials in Europe but failed FDA-approval and treatment in mice has given contrasting outcomes [51], [57]. Targeting MMP via doxycycline has also been used, while inhibition of PLC/IP_3_/PKC/ERK reduced lethality and aortic rupture. Similarly, targeting androgen signalling was also effective. The cross talk between these pathways, how they are activated by either ECM defects and/or the impact of altered proteostasis, remains poorly defined (AR: androgen receptor, Hsp heat shock protein).

Recent elegant work by Dietz and colleagues has suggested a key role for increased signalling of the PLC/IP_3_/PKC/ERK pathway which was identified following transcriptomics of thoracic aorta of mice with a *Col3a1* glycine mutation (*Col3a1^G209S/+^* and *Col3a1^G938D/+^*) (Fig. 2, Fig. 3) [51]. Inhibition of this pathway increased the survival of these mice, confirming its contribution to disease. However, how this pathway is activated and the mechanism by which it causes disease remains unclear. The transcriptomic analysis did not show overt signs of UPR activation although ER enlargement was detected, and the reduced levels of collagen III were therefore put forward as the upstream mechanism [51]. Confirmation in the *Col3a1* knock-out models would be highly informative in this regard and further analysis is required to disentangle this cross-talk.

Pregnancy associated aortic rupture is the most common cause of death for female vEDS patients of child bearing age [95]. The PLC/IP_3_/PKC/ERK signalling pathway was also increased during pregnancy and lactation [51], which also occurs in Marfan Syndrome where this is due to oxytocin signalling [96]. This potentially convergent mechanism between Marfan Syndrome and vEDS was confirmed by the 95% survival of pregnant *Col3a1^G209S^*^/+^ mice treated by an oxytocin receptor antagonist and when pups were removed immediately after birth [51]. This clearly highlights the increased risk of lactation for vEDS in the mother and the link between oxytocin signalling,- PLC/IP_3_/PKC/ERK signalling and vEDS. Together this shows that over-activation of the PLC/IP_3_/PKC/ERK pathway in both non pregnant and pregnant females contributes to aortic rupture, underscoring a key role for this signalling pathways in vEDS (Fig. 3). There is also cross-talk with this PLC/IP_3_/PKC/ERK pathway and androgen signalling. Data from patients indicate that males are more severely affected than females, which is also reflected in the lethality of the mouse models, that was rescued when treated with bicalutamide, an androgen receptor antagonist [51] (Fig. 3).

## Therapeutic approaches

Current treatments of vEDS are only symptomatic due to our limited understanding of its disease mechanism. To date, the main treatment is to maintain normal blood pressure to reduce the likelihood of vascular dissection or rupture, via angiotensin receptor blockers, ß-adrenergic blockers or other antihypertensive agents. Blood pressure medication has also been employed in mouse models. Two mouse models were treated with anti-hypertensive drugs losartan and propranolol but while they reduced the blood pressure, no changes were seen in the incidence of the vascular ruptures and survival of the mice [51], [57]. These data support that reducing blood pressure may be ineffective for preventing vascular ruptures in mouse models.

To date, only one clinical trial was done using celiprolol, a ß1 antagonist and ß2 agonist that reduces arterial pressure [97]. Patients treated with celiprolol had vascular complications later than untreated patients [97], and a subsequent long-term observational study also suggested a beneficial effect on survival compared to published data [98]. However due to design of the observational study (absence of untreated arm) the high survival cannot be unequivocally attributed to celiprolol [98]. The original trial was also criticised for its small sample size and that one third of patients did not have a *COL3A1* mutation, complicating its interpretation [36]. Furthermore, whilst celiprolol has some efficacy, it is not well tolerated (1/3 patients did not tolerate recommended dose) [99] and was declined FDA approval. Interestingly in mice, celiprolol rescued the biomechanical parameters of one vEDS model [57], however treatment with a different ß blocker (bisoprolol) did not have the same benefit [100] (Fig. 3). This raises the question regarding the mechanism of celiprolol, and confirms that ß blockers cannot be used interchangeably, which is relevant as celiprolol is not available in the USA and Canada, but is in Europe [100]. Moreover, treatment with celiprolol gave opposing results when administered to two different mouse models and in one increased the levels of aortic ruptures and death [51]. Importantly, these two studies provide evidence for allele specific treatment outcomes. The basis of this allelic heterogeneity remains unclear (e.g. was this due to genetic background or different alleles?) but illustrates the potential problems of targeting downstream effects of mutations, and that there is an urgent need to delineate disease mechanisms for different classes of *COL3A1* mutations and in multiple disease relevant cell types.

The identification of the PLC/IP_3_/PKC/ERK pathway in two different mouse models with glycine mutations [51] presents an important advance in our knowledge of vEDS and development of therapeutic opportunities. This is especially so given the cross talk with androgen and oxytocin signalling that contributes to the lethality during pregnancy, following lactation, and puberty [51]. Extensive modulation of this pathway via ruboxistaurin (PKCβ inhibition), cobimetinib (FDA-approved inhibitor of MEK, the kinase that activates ERK), and hydralazine (FDA-approved, inhibits IP_3_-mediated calcium release from ER and PKCβ activation) shows its potential as a therapeutic target [51] (Fig. 3). It should be noted that the protection of hydralazine was lost around puberty, especially in males but that a combined treatment with the FDA-approved compound spironolactone (androgen receptor antagonist) overcame this lethality [51]. These are highly promising results and have uncovered a key pathway and potential therapeutic target in vEDS. However and importantly, this investigation was focused only on the lethality and aortic dissection, and did not consider extra-vascular phenotypes.

Finally, attention has also been focused on directly modulating the ECM in vEDS. In particular, as *Col3a1*^+/−^ mice exhibited elevated levels of MMP9 [73], treatment with broad spectrum MMP inhibitor doxycycline was undertaken (both as a short term, and long term chronic treatment) which reduced aortic lesions [72], [73] (Fig. 3). This suggests MMPs could be a treatment target at least for nonsense mutations leading to haploinsufficiency. Further analysis is now needed to confirm if these data and approach can be translated to other mutations.

Besides pharmacological approaches, gene therapy strategies offer the potential of, at least in theory, providing a cure by addressing the root-cause of the disease. One of these is RNA interference to knock-down the expression of the mutant *COL3A1* allele. A proof of concept of this approach has been performed in fibroblasts from vEDS patients in which the mutant *COL3A1* mRNA was successfully targeted without affecting the WT allele [79]. This improved collagen fibril formation, further confirming that the fibril defects are at least in part due to insufficient extracellular collagen III. In addition, a very limited analysis showed reduction of a single ER stress marker [79]. While this is promising, for this technology to be adopted it requires successful allelic specific knockdown for a variety of alleles. In addition, given the multi-systemic nature of vEDS, the efficient delivery of any RNAi to the different tissues and cells represents a hurdle for the development of this approach as a treatment.

## Concluding remarks

The disease mechanisms of *COL3A1* mutations and vEDS remain enigmatic and a complex puzzle to solve. However, by combining elegant animal models that accurately recapitulate the genetic and phenotypic features of vEDS with global analysis of cell responses to these mutations, significant advances have been made. They however have also uncovered complexities. At least some of these pertain to the molecular basis of the allelic and mechanistic heterogeneity across the different cell types affected by mutations in *COL3A1*. As a result it is unlikely, but not impossible, that targeting downstream mechanism can lead to a one size fits all treatment for vEDS. An alternative or complementary approach would be a causal treatment whereby the upstream mechanisms of reduced collagen levels and ER retention are tackled, and/or the use of gene therapy approaches as a cure. Regardless of approach, the multi-systemic nature of the disease does mean that delivery will need to be carefully considered. The available mouse models (and possibly future generation of additional novel models) for vEDS are very valuable resources for the study of this disease and our quest for disease-specific (and/or personalized) therapies, the ultimate aim being increased life-expectancy and quality of life of those living with this severe disease.

## CRediT authorship contribution statement

**Ramla Omar:** conceptualization, Writing original draft, writing- reviewing and editing. **Fransiska Malfait:** funding acquisition, conceptualization, Writing original draft, writing- reviewing and editing. **Tom Van Agtmael:** funding acquisition, conceptualization, Writing original draft, writing- reviewing and editing.

## Declaration of Competing Interest

The authors declare that they have no known competing financial interests or personal relationships that could have appeared to influence the work reported in this paper.
